# Chicken or egg? The evolutionary riddle of CMS and *Rf* in plants

**DOI:** 10.1111/jipb.70292

**Published:** 2026-05-13

**Authors:** Peilin Wang, Zhe Zhao, Qibin Wu, Yaoguang Liu, Youxiong Que

**Affiliations:** ^1^ State Key Laboratory of Tropical Crop Breeding, Institute of Tropical Bioscience and Biotechnology Chinese Academy of Tropical Agricultural Sciences Sanya 572024 China; ^2^ Guangdong Laboratory for Lingnan Modern Agriculture, Guangdong Basic Research Center of Excellence for Precise Breeding of Future Crops, College of Agriculture South China Agricultural University Guangzhou 510642 China

## Abstract

Cytoplasmic male sterility (CMS) reduces pollen fertility and nuclear Rf genes restore fertility. This Commentary explores support for the “arms race” and “pre‐adaptation” models for CMS–*Rf* co‐evolution. CMS and Rf interact dynamically, influencing plant reproduction, stress tolerance, and hybrid seed production, highlighting co‐evolutionary processes critical for crop improvement.

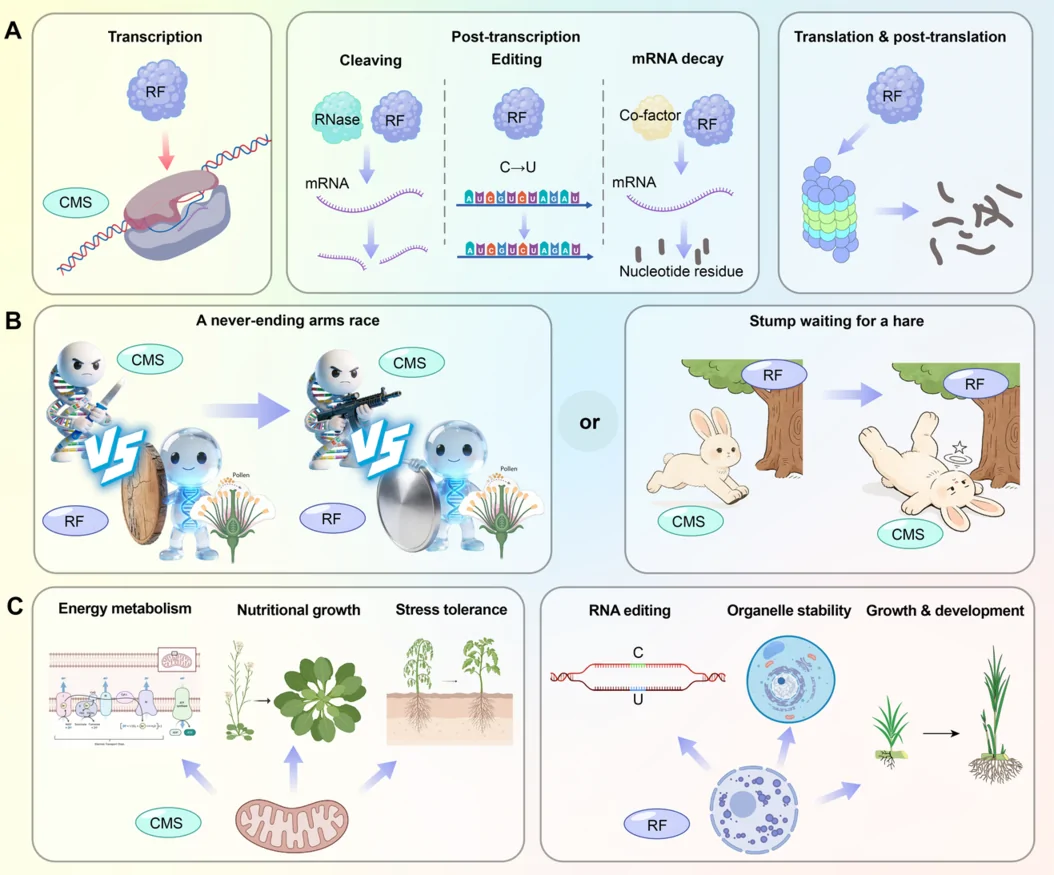

Cytoplasmic male sterility (CMS) is a maternally inherited trait caused by aberrant mitochondrial genes (CMS genes) whose toxic products interfere with anther/pollen metabolism (Luo et al., 2013; Chen and Liu, 2014; Tang et al., 2017). Nuclear *restorer‐of‐fertility* (*Rf*) genes, predominantly encoding Pentatricopeptide repeat (PPR) proteins (sequence‐specific RNA‐binding factors), suppress CMS via transcriptional, post‐transcriptional, translational, or post‐translational regulation (Wang et al., 2006; Luo et al., 2013; Tang et al., 2014; Wang and Tan, 2025; Figure 1). This system exemplifies an intrinsic genomic conflict: mitochondrial genes are selected for traits that inhibit male gametogenesis and preserve resources, whereas nuclear genes are selected to promote fitness through both male and female reproductive pathways (Wang and Tan, 2025).

**Figure 1:**
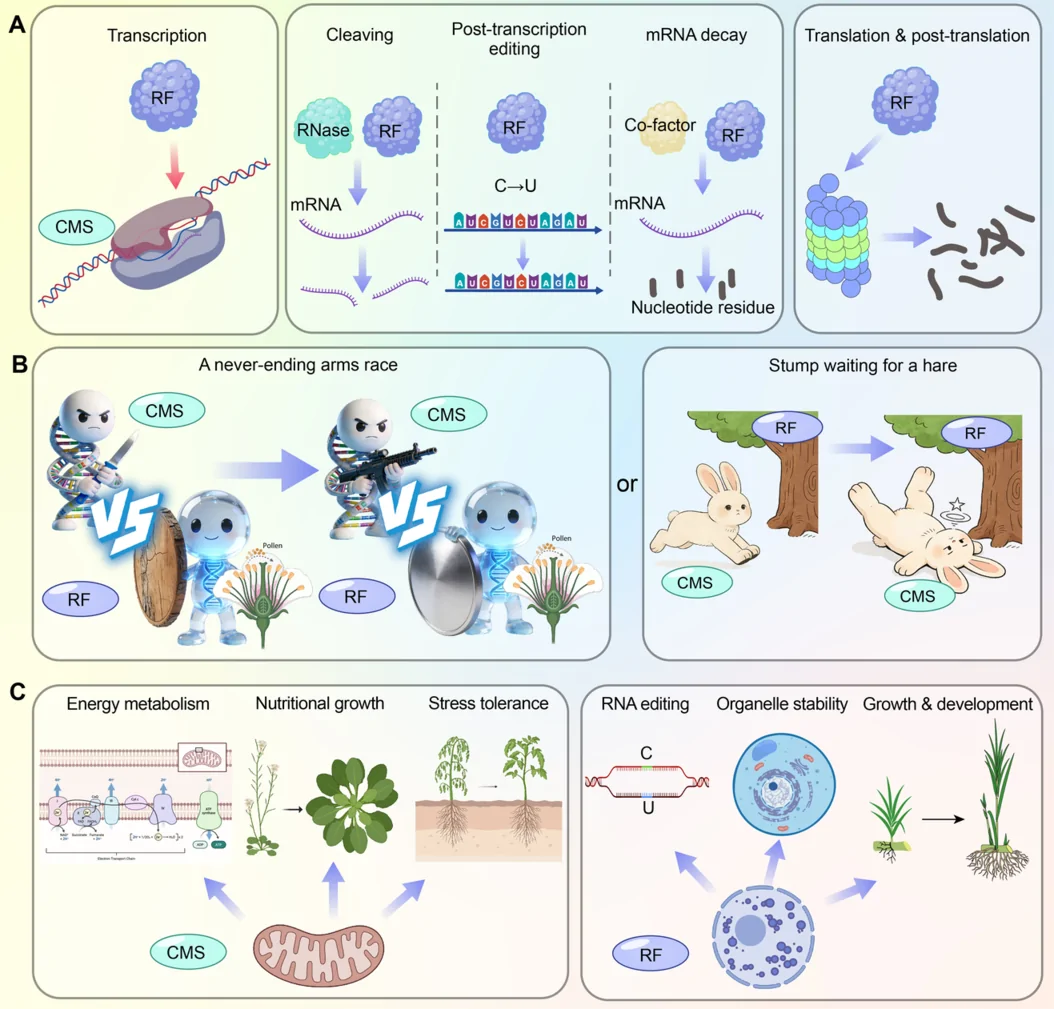
The evolutionary riddle of CMS and *Rf* in plants **(A)** Restorer genes mediate fertility restoration of CMS from transcriptional, post‐transcriptional, translational, and post‐translational levels. **(B)** The evolutionary order of CMS and *Rf*: A never‐ending “arms race” or the “stump‐waiting‐for‐a hare”. **(C)** The potential functions of CMS and RF.

The evolutionary order of CMS and *Rf* genes has long been a riddle, with “Arms Race” and “Pre‐adaptation” hypotheses that reflect distinct modes of co‐evolution (Figure 1). These two hypotheses are not mutually exclusive: Some CMS‐*Rf* pairs may arise through arms race co‐evolution, while others reflect pre‐adaptation. Resolving this riddle requires integrating comparative genomics, for example, tracing the origins of CMS and *Rf* genes in phylogenetic lineages, or functional analyses such as determining the ancestral functions of *Rf* genes.

The dominant hypothesis is “Arms Race”. It posits that CMS genes arise first through mitochondrial mutations, and *Rf* genes then evolve under the selective pressure imposed by CMS. This antagonistic co‐evolution resembles a “Red Queen” dynamic (a concept from evolutionary biology describing an endless struggle to maintain fitness relative to a co‐evolving antagonist), where each genome must continuously adapt to counteract the other. During this process, mitochondrial genomes accumulate new mutations to generate more potent CMS genes that evade existing *Rf* suppressors, while nuclear genomes evolve novel *Rf* alleles or loci to recognize and silence these new CMS variants, most often via PPR gene duplication and divergence (Chen and Liu, 2014).

Evidence supporting this hypothesis comes from the rapid diversification of both CMS‐associated mitochondrial genes and their corresponding nuclear *Rf* genes among closely related species. Rice harbors multiple CMS types (WA, BT, HL, and FA) with distinct mitochondrial determinants, each associated with a specific set of *Rf* genes (Table S1). For example, in rice, the divergent members (i.e., *Rf1a/Rf5*, *Rf1b*, *Rf4*) of the PPR gene cluster on Chromosome 10 have evolved to suppress different CMS genes (i.e., *orf79*, *orfH79*, *WA352*) (Tang et al., 2014). In radish Ogura CMS, the restorer protein recognizes *orf138* transcripts through specific binding sites, and the sequence variation in both the *Rf* locus and the CMS‐associated mitochondrial locus jointly shapes restoration efficiency and specificity—providing strong support for CMS‐driven selection on *Rf* evolution. More broadly, the PPR gene family exhibits high rates of duplication and signatures of positive selection, consistent with adaptive evolution under CMS‐driven selective pressure (Colcombet et al., 2013; Wang and Tan, 2025). Evolutionary rates further support this arms‐race scenario. That is, Rf‐like PPR genes across angiosperms exhibit significantly elevated nonsynonymous‐to‐synonymous substitution ratios (dN/dS) compared to other PPR genes, a hallmark of diversifying selection driven by antagonistic nuclear‐mitochondrial conflicts. Notably, the positively selected residues map precisely to the RNA‐binding surface of PPR motifs, suggesting that recurrent selection pressure from evolving CMS transcripts continuously reshapes the recognition specificity of Rf proteins.

An alternative scenario is the “Pre‐adaptation” hypothesis, suggesting that *Rf* genes existed before the emergence of CMS, serving ancestral functions in mitochondrial RNA metabolism, for example, cleaving, editing, or stability control. When a new CMS mutation arises, these pre‐existing nuclear genes accidentally gain the ability to suppress the CMS phenotype—a phenomenon analogous to a Chinese parable describing an accidental success: “waiting by the stump for a hare”.

This hypothesis is supported by the pleiotropic functions of several *Rf* genes. For example, Arabidopsis PPR proteins involved in mitochondrial RNA editing can suppress CMS when introduced into CMS lines of other species, suggesting their ancestral role in organellar RNA processing (Colcombet et al., 2013). Similarly, the rice *Rf4* gene variants, including functional and non‐functional forms, exist in ancient wild rice species. Because *WA352c* appears to be a relatively recently evolved mitochondrial sterility gene in *Oryza rufipogon*, the more ancient *Rf4* and its variants may have served other, as‐yet‐undiscovered intrinsic functions before being co‐opted—or neofunctionalized—as a fertility restorer for *WA352c* during the adaptive evolution of wild rice (Tang et al., 2017). The maize *Rf2* gene also plays a role in normal mitochondrial metabolism, as loss‐of‐function mutations cause developmental defects even in the absence of CMS (Chen and Liu, 2014). These observations imply that some *Rf* genes are co‐opted from core mitochondrial regulatory networks to counteract CMS, rather than evolving *de novo*.

CMS reduces male fertility, yet it persists in diverse plant lineages, suggesting that it confers adaptive advantages beyond genomic conflict, or in other words, CMS is retained in nature for ecological and pleiotropic benefits.

Interestingly, CMS and *Rf* genes are maintained in dynamic equilibrium through frequency‐dependent selection. When CMS is rare, CMS plants benefit from abundant pollen provided by fertile (*Rf*‐carrying) individuals, allocating more resources to seed production. As CMS becomes more common, fertile plants gain a selective advantage by contributing disproportionately to pollen pools, increasing the frequency of *Rf* genes. These cyclical balances prevent the fixation of either CMS or *Rf* alleles, maintaining genetic polymorphism in natural plant populations. On the other hand, CMS genes often exert pleiotropic effects on plant performance, particularly in stress tolerance (Wang et al., 2013; Xiao et al., 2023). Conversely, these benefits may offset the cost of male sterility, explaining the retention of CMS in highly stressful environments.

*Rf* genes, particularly PPR‐type, are not specialized solely for CMS suppression but are also integral components of organellar RNA regulatory networks (Wang and Tan, 2025). Their pleiotropic functions have important implications for plant development and agronomic performance. As reported, PPR proteins encoded by *Rf* genes regulate the expression of numerous mitochondrial genes involved in energy metabolism, respiration, and stress responses (Colcombet et al., 2013). Additionally, the RNA‐binding promiscuity of PPR proteins raises the possibility of off‐target effects. That is, an RF protein targeting a CMS transcript may inadvertently bind to other mitochondrial mRNAs and disrupt their processing or stability (Wang and Tan, 2025). The off‐target effects could explain the developmental defects observed in some *Rf*‐overexpressing lines.

The CMS/*Rf* system remains an invaluable tool in hybrid seed production and agricultural biotechnology. However, the evolutionary dynamics between CMS and *Rf* genes continue to present challenges for long‐term sustainability. The emergence of new CMS variants that evade existing *Rf* genes, as well as the potential for linkage drag associated with *Rf* loci, requires ongoing investigation. Despite these challenges, the CMS/*Rf* system provides immense opportunities for crop improvement and plant reproductive biology.

The unresolved question of whether CMS or *Rf* genes evolved first remains a topic of interest. Investigating these evolutionary dynamics through co‐evolutionary models could provide critical insights into how mitochondrial and nuclear genomes interact and adapt. Comparative genomics and evolutionary simulations could help track the evolutionary history of CMS and *Rf* genes, shedding light on how they shape reproductive strategies in plants. Future studies should also explore the potential existence of ancient *Rf* genes that predate CMS mutations and examine how these genes may have co‐evolved with mitochondrial dysfunctions to influence reproductive success across diverse plant species.

The PPR proteins' unintended interactions with non‐CMS mitochondrial transcripts could disrupt other mitochondrial processes, causing developmental defects. The future of *Rf* gene engineering could involve using PPR proteins as next‐generation plant endogenous RNA‐editing tools (Manavski et al., 2025). By enhancing the specificity of these proteins for their target CMS transcripts, we could minimize off‐target effects and create highly precise, targeted RNA‐editing tools for plants. Additionally, advanced gene‐editing technologies could be employed to engineer PPR proteins that function as programmable RNA‐editing tools, allowing for precise manipulation of plant mitochondrial transcripts with minimal collateral effects. This approach would enable the creation of plants with tailored traits, further improving the efficiency and resilience of hybrid seed production systems.

CMS lines, despite their male sterility, often exhibit improved tolerance to abiotic stresses such as drought and salinity. This could be due to the altered mitochondrial respiration and reactive oxygen species (ROS) scavenging mechanisms in CMS plants (Xiao et al., 2023). Similarly, *Rf* genes, particularly PPR proteins, may also influence mitochondrial metabolism, providing plants with a greater ability to adapt to environmental stressors during reproductive development. Future research should explore the role of CMS and *Rf* genes in mediating stress tolerance during the reproductive phase. The potential for targeted application of CMS and *Rf* genes to enhance reproductive success under stressful conditions could lead to the development of crops that are not only more resilient but also more productive in challenging environments.

In conclusion, the CMS/*Rf* system provides a window into the complex molecular and evolutionary processes that shape plant reproduction. By focusing on co‐evolutionary dynamics, refining gene‐editing technologies, and exploring the pleiotropic roles of CMS and *Rf* genes, future research will not only advance our understanding of plant biology but also enhance the practical applications of these systems in sustainable agriculture. Up to this day, whether the “chicken” (*Rf*) precedes the “egg” (CMS) or vice versa remains an open question, but their intertwined histories illuminate how plant nuclear and cytoplasmic genomes continually negotiate harmony through perpetual conflict—a biological dialogue as ancient as reproduction itself.

## CONFLICTS OF INTEREST

The authors declare no conflicts of interest.

## AUTHOR CONTRIBUTIONS

W.P., L.Y., and Q.Y. conceived and designed the study. W.P. wrote the original draft. W.P., W.Q., Z.Z., L.Y., and Q.Y. reviewed and edited the manuscript. W.P., Q.Y., and Z.Z. acquired the funding. All authors read and approved the final manuscript.

## Supporting information

Additional Supporting Information may be found online in the supporting information tab for this article: http://onlinelibrary.wiley.com/doi/10.1111/jipb.70292/suppinfo

Table S1. Some representation Rf genes characterized in plant
