# Supplementary material for: Chicken or egg? The evolutionary riddle of CMS and Rf in plants
Source: J Integr Plant Biol. 2026 May 13;68(10):3540–2. doi: 10.1111/jipb.70292 (PMC13629266; doi:10.1111/jipb.70292)
Supplement: Supplementary file 1 [file JIPB-68-3540-s001.doc]

| **Species** | **CMS type** | ***Rf* gene** | **PPR** | **Reference** |
| --- | --- | --- | --- | --- |
| *Zea mays* | CMS-S (G) | *Rf3* | *√* | (Qin et al., 2021) |
| *Oryza sativa* | CMS-BT (G) | *Rf1a (R), Rf1b (R)* | *√* | (Wang et al., 2006) |
|  | CMS-HL (G) | *Rf5* | *√* | (Hu et al., 2012; Wang et al., 2013) |
|  | CMS-HL (G) | *Rf6* | *√* | (Huang et al., 2015) |
|  | CMS-WA (S) | *Rf4* | *√* | (Tang et al., 2014) |
|  | CMS-WA (S) | *Rf20* | *√* | (Song et al., 2024) |
|  | CMS-FA | *OsRf19* | *√* | (Jiang et al., 2022) |
| *Brassica napus* | CMS-Ogu (S) | *PPR-B* | *√* | (Uyttewaal et al., 2008) |
| *Sorghum bicolor* | CMS-A1 (G) | *Rf1, Rf2* | *√* | (Klein et al., 2005; Jordan et al., 2010) |
| *Petunia* | CMS | *PPR592* | *√* | (Bentolila et al., 2002) |
| *Raphanus sativus* | CMS-Kos (S) | *Rfk1* | *√* | (Koizuka et al., 2003) |
| *Triticum aestivum* | CMS-T | *Rf1, Rf3* | *√* | (Melonek et al., 2021) |
| *Gossypium hirsutum* | CMS-D2 | *D05_PPR-cluster* | *√* | (Gao et al., 2022) |
| *Beta vulgaris* | CMS-Owen | *Rf1* | *×* | (Matsuhira et al., 2012; Kitazaki et al., 2015) |
| *Phaseolus vulgaris* | CMS | *Fr* | *×* | (He et al., 1995) |

Table S1. Some representation Rf genes characterized in plant

**References**

Hu J, Wang K, Huang W, et al. The rice pentatricopeptide repeat protein RF5 restores fertility in Hong-Lian cytoplasmic male-sterile lines via a complex with the glycine-rich protein GRP162. Plant Cell. 2012;24(1):109-122.

Huang W, Yu C, Hu J, et al. Pentatricopeptide-repeat family protein RF6 functions with hexokinase 6 to rescue rice cytoplasmic male sterility. Proc Natl Acad Sci U S A. 2015;112(48):14984-14989.

Jiang H, Lu Q, Qiu S, et al. Fujian cytoplasmic male sterility and the fertility restorer gene *OsRf19* provide a promising breeding system for hybrid rice. Proc Natl Acad Sci U S A. 2022;119(34):e2208759119.

Jordan DR, Mace ES, Henzell RG, Klein PE, Klein RR. Molecular mapping and candidate gene identification of the *Rf2* gene for pollen fertility restoration in sorghum [*Sorghum bicolor* (L.) Moench]. Theor Appl Genet. 2010;120(7):1279-1287.

Klein RR, Klein PE, Mullet JE, Minx P, Rooney WL, Schertz KF. Fertility restorer locus Rf1 [corrected] of sorghum (*Sorghum bicolor* L.) encodes a pentatricopeptide repeat protein not present in the colinear region of rice chromosome 12. Theor Appl Genet. 2005;111(6):994-1012.

Qin X, Tian S, Zhang W, et al. The main restorer *Rf3* of maize S type cytoplasmic male sterility encodes a PPR protein that functions in reduction of the transcripts of orf355. Mol Plant. 2021;14(12):1961-1964.

Song S, Li Y, Qiu M, et al. Structural variations of a new fertility restorer gene, *Rf20*, underlie the restoration of wild abortive-type cytoplasmic male sterility in rice. Mol Plant. 2024;17(8):1272-1288.

Bentolila S, Alfonso AA, Hanson MR. A pentatricopeptide repeat-containing gene restores fertility to cytoplasmic male-sterile plants. Proc Natl Acad Sci U S A. 2002;99(16):10887-10892. doi:10.1073/pnas.102301599

Tang H, Luo D, Zhou D, et al. The rice restorer *Rf4* for wild-abortive cytoplasmic male sterility encodes a mitochondrial-localized PPR protein that functions in reduction of WA352 transcripts. Mol Plant. 2014;7(9):1497-1500.

Uyttewaal M, Arnal N, Quadrado M, et al. Characterization of *Raphanus sativus* pentatricopeptide repeat proteins encoded by the fertility restorer locus for *Ogura* cytoplasmic male sterility. Plant Cell. 2008;20(12):3331-3345.

Wang Z, Zou Y, Li X, et al. Cytoplasmic male sterility of rice with boro II cytoplasm is caused by a cytotoxic peptide and is restored by two related PPR motif genes via distinct modes of mRNA silencing. Plant Cell. 2006;18(3):676-687.

Koizuka N, Imai R, Fujimoto H, et al. Genetic characterization of a pentatricopeptide repeat protein gene, *orf687*, that restores fertility in the cytoplasmic male-sterile *Kosena radish*. Plant J. 2003;34(4):407-415.

He S, Yu ZH, Vallejos CE, Mackenzie SA. Pollen fertility restoration by nuclear gene *Fr* in CMS common bean: an *Fr* linkage map and the mode of *Fr* action. Theor Appl Genet. 1995;90(7-8):1056-1062.

Kitazaki K, Arakawa T, Matsunaga M, et al. Post-translational mechanisms are associated with fertility restoration of cytoplasmic male sterility in sugar beet (*Beta vulgaris*). Plant J. 2015;83(2):290-299.

Matsuhira H, Kagami H, Kurata M, et al. Unusual and typical features of a novel *restorer*-*of*-*fertility* gene of sugar beet (*Beta vulgaris* L.). Genetics. 2012;192(4):1347-1358.

Melonek J, Duarte J, Martin J, et al. The genetic basis of cytoplasmic male sterility and fertility restoration in wheat. Nat Commun. 2021;12(1):1036.

Gao B, Ren G, Wen T, Li H, Zhang X, Lin Z. A super PPR cluster for restoring fertility revealed by genetic mapping, homocap-seq and de novo assembly in cotton. Theor Appl Genet. 2022;135(2):637-652.
